# *Schistosoma japonicum* Soluble Egg Antigens Attenuate IFN-γ-Induced MHC Class II Expression in RAW 264.7 Macrophages

**DOI:** 10.1371/journal.pone.0049234

**Published:** 2012-11-13

**Authors:** Gui-Xia Tang, He-Jun Zhou, Jin-Wei Xu, Jin-Mei Xu, Min-Jun Ji, Hai-Wei Wu, Guan-Ling Wu

**Affiliations:** 1 Department of Pathogen Biology and Immunology, Nanjing Medical University, Nanjing, Jiangsu, China; 2 Jiangsu Province Key Laboratory of Modern Pathogen Biology, Nanjing, Jiangsu, China; 3 Center for International Health Research, Rhode Island Hospital, Providence, Rhode Island, United States of America; 4 National Institute of Parasitic Diseases, Chinese Center for Disease Control and Prevention; Key Laboratory of Parasite and Vector Biology, MOH, China, WHO Collaborating Centre for Malaria, Schistosomiasis and Filariasis, Shanghai, China; 5 MacroStat Clinical Research Co. Ltd, Shanghai, China; Queensland Institute of Medical Research, Australia

## Abstract

Innate immune response plays the key role in initiating and guiding the immune response. Elucidating the innate immune related molecular events involved in the interaction between the parasite and the host will aid in the development of an effective vaccine and anti-schistosome pharmaceuticals. In this study, we examined the regulatory effect of *Schistosoma japonicum* soluble egg antigen (SEA) on MHC class II expression in macrophage cell line RAW 264.7. We demonstrated that SEA possesses the ability to down-regulate IFN-γ-induced MHC class II expression in RAW 264.7 cells. The production of IL-10 and IL-6 in RAW 264.7 cells, induced by SEA, was responsible for mediating the down-regulation of MHC class II. Our findings suggest that in RAW 264.7 cells (1) IFN-γ provides a condition for lower concentrations of SEA to attenuate MHC class II expression; (2) SEA attenuated IFN-γ-induced MHC class II expression and the IL-10 and IL-6 production is mediated at least partly by the interaction of SEA with TLR4; and (3) SEA attenuated IFN-γ-induced MHC class II expression at the transcriptional level.

## Introduction

Schistosomiasis continues to be one of the major health problems in the developing world since the control strategy centered on mass chemotherapy has failed to effectively control this disease. Currently, more than 207 million people worldwide are infected with schistosomiasis [1]. Therefore, new and more effective control strategies including vaccines are urgently needed. Toward this end, trying to clarify the molecular events involved in the interaction between schistosome and immunocytes is crucial. Most studies on the immunology of schistosome infection have focused on the molecular mechanisms of adaptive immune responses. In view of the important role of innate immunity in initiating and regulating acquired immunity [2], it is necessary to study the impact of innate immunity on schistosome infection, including the relationship between schistosome antigens and antigen-presenting cells.

The expression of MHC class II is affected by infection. Many pathogens such as *Mycobacterium*, *Murine cytomegalovirus* and *Leishmania donovani* possess the ability to suppress MHC class II expression and use it as a means of evading the host’s immunological attack [3], [4], [5]. Some studies suggested that several components of schistosome SEA or SEA-induced factors might play a regulatory role on the I-A expression in granuloma macrophages (GMs) [6]. Based on the key role of MHC class II in presenting foreign antigen to T cells, the limitation of MHC class II expression might represent one of the most effective strategies for down-regulating immune responses leading to evasion of the parasite from host’s immune attack.

Following the deposition of schistosome eggs in the tissue affected and the massive release of egg antigen, intense immune response is aroused indicating the development of the acute phase of schistosomiasis [7]. Despite the uninterrupted antigenic stimulation, a down regulation of the granulomatous response is observed in chronic schistosomiasis [8]. The response events involved in acquired immunity in this course have acquired a broad exploration but no more studies were focused on the MHC class II expression on antigen presenting cells regulated by schistosome-derived components. Macrophages are the main antigen presenting cells presenting in schistosome egg granulomas, account for about 30% of the total cells in egg granulomas [9]. We thus selected macrophage cell line RAW 264.7 in this study for detail exploration of the regulatory effect of SEA on MHC class II expression.

## Results

### SEA Attenuated IFN-γ-induced MHC Class II Expression in RAW 264.7 Cells

**Figure 1:**
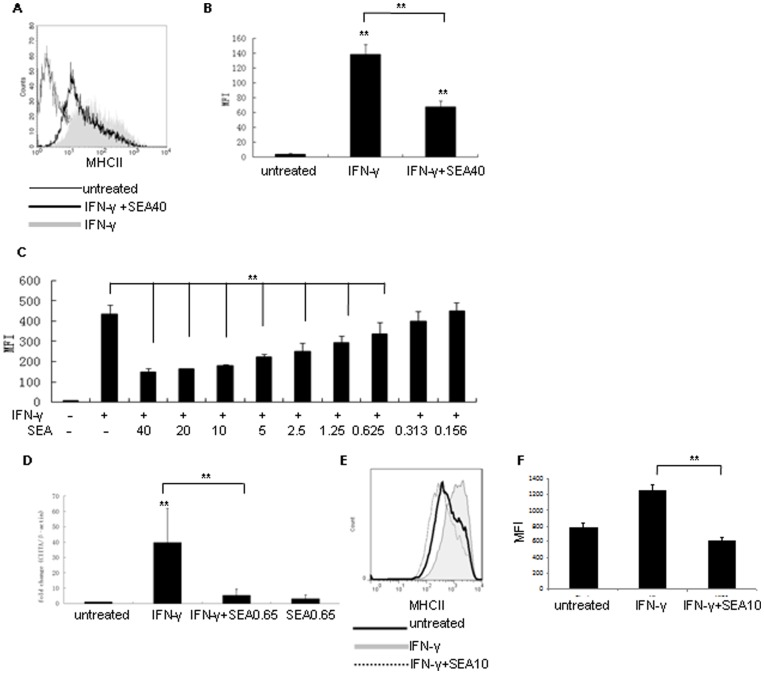
SEA attenuated MHC class II expression in RAW 264.7 cells. (A) Flow cytometric analysis of MHC class II expression in RAW 264.7 cells stimulated with IFN-γ (shaded histogram), 40 µg/ml of SEA (SEA40) and IFN-γ simultaneously (open histogram with thick line) and untreated cells (open histogram with thin line). (B) Histogram showing the mean fluorescence intensity (MFI) of MHC class II expression in RAW 264.7 cells stimulated with IFN-γ, 40 µg/ml of SEA (SEA40) and IFN-γ simultaneously and cells cultured with medium alone (untreated). (C) Histogram showing the mean fluorescence intensity (MFI) of MHC class II expression in RAW 264.7 cells stimulated with IFN-γ and serially diluted SEA from 40 µg/ml to 0.156 µg/ml simultaneously, or with IFN-γ alone, or with medium alone for 48 h. (D) Fold change of CIITA mRNA relative to β-actin mRNA in RAW 264.7 cells stimulated with IFN-γ, 0.65 µg/ml SEA (SEA0.65), or IFN-γ and 0.65 µg/ml SEA simultaneously (IFN-γ + SEA0.65), cells cultured with medium alone (untreated) were used as control. (E) Flow cytometric analysis of MHC class II expression in RAW 264.7 cells stimulated with IFN-γ (R&D) (shaded histogram), 10 µg/ml of SEA (SEA10, treated with endotoxin removing gel, endotoxin level is 0 EU/ml) and IFN-γ simultaneously (open histogram with dotted line) and untreated cells (open histogram with thick line). (F) Histogram showing the mean fluorescence intensity (MFI) of MHC class II expression in RAW 264.7 cells stimulated with 10 ng/ml IFN-γ (R&D), 10 µg/ml of SEA (SEA10, treated with endotoxin removing gel, endotoxin level is 0 EU/ml) and IFN-γ simultaneously and cells cultured with medium alone (untreated). (**P<0.01).

IFN-γ, which has multiple immunomodulatory roles in immune responses, is a very effective cytokine for up-regulating MHC class II expression and is necessary for the host’s defense to pathogens [10]. Macrophages are one of the most important cells influenced by IFN-γ. IFN-γ can directly promote antigen processing and presenting ability of macrophages [10]. To evaluate the regulatory role of SEA on MHC class II, we selected IFN-γ as an inducer of MHC class II expression so that we can make a detailed observation of the effect of SEA on regulating MHC class II expression. To ascertain the appropriate concentration of IFN-γ used for up-regulation of MHC class II, RAW 264.7 cells were incubated with serially diluted IFN-γ for 48 h. 4 ng/ml IFN-γ (5×10^6^ IU/mg, PeproTech) was the lowest effective dose for significantly upregulating MHC class II in RAW 264.7 cells and was selected for use throughout the study (data not shown). Compared with MHC class II expression in RAW264.7 cells incubated with IFN-γ alone, 40 µg/ml SEA can significantly attenuated IFN-γ-induced MHC class II expression in RAW 264.7 cells (*P*<0.01). (Figure 1A, 1B). Considering the amount of deposited eggs increased gradually with the elongation of the worms′life in vivo or with the development of schistosomiasis, we analyzed the ability of different concentrations (serially diluted from 40 µg/ml to 0.156 µg/ml) of SEA at attenuating IFN-γ-induced MHC class II expression. The results showed that SEA significantly attenuated IFN-γ-induced MHC class II expression in RAW 264.7 cells in a dose-dependent fashion between concentration changing from 40 µg/ml to 0.625 µg/ml (Figure 1C) (*P*<0.01), and this effect was lost at concentrations of 0.313 µg/ml and 0.156 µg/ml.

CIITA, the MHC class II transactivator protein, is involved in the inducible expression of class II genes upon IFN-γ treatment and is controlled and induced by IFN-γ [11]. To analyze whether MHC class II expression was attenuated by SEA at the transcriptional level, we tested the level of CIITA by real-time PCR. RAW 264.7 cells were primed with 0.65 µg/ml of SEA in the presence or absence of IFN-γ. Cells primed with IFN-γ or culture medium alone (untreated) were used as controls. CIITA mRNA transcriptional level in these cells were determined after 48 h of culture. The results indicated that the transcription of CIITA induced by IFN-γ was attenuated significantly by SEA (Figure 1D). This suggested that the attenuation of IFN-γ-induced MHC class II expression by SEA might happened at transcriptional level.

To exclude the possible effects of contaminated endotoxin in the prepared SEA on MHC class II expression, we pretreated the SEA with Detoxi-Gel™ Endotoxin Removing Gel (Thermo Scientific Pierce) and then tested the activity of endotoxin contained in the treated SEA sample. The results showed that following the treatment with endotoxin removing gel, the activity of endotoxin contained in the prepared SEA is similar to the negative control (0 EU/ml). Then RAW 264.7 cells were treated with 10 ng/80 IU/ml IFN-γ (R&D) in presence or absence of 10 µg/ml SEA for 48 h at 37°C, 5% CO_2._ Cells cultured in medium alone were used as untreated control. The results showed that these SEA still possesses the effects in significantly attenuating IFN-γ-induced MHC class II expression in RAW 264.7 cells (Figure 1E and 1F).

### The Production of IL-10 and IL-6 Induced by SEA from RAW 264.7 Cells were Promoted by IFN-γ

The granulomas formation around the deposited schistosome eggs in liver is dependent on type IV hypersensitivity reaction which depends on effective antigen presentation. Our results above showed that SEA could down-regulate the expression of MHC class II induced by IFN-γ (Figure 1B). IL-10 and IL-6 have been reported to possess the ability in down-regulating the expression of MHC class II through feedback effects [12], [13] and are induced following the deposition of schistosome eggs in vivo [14].

**Figure 2:**
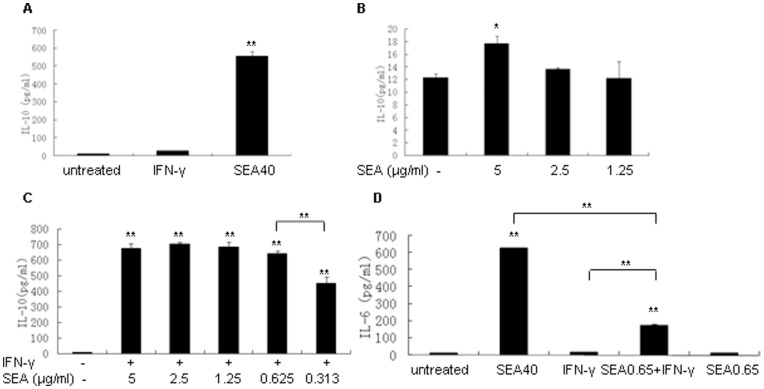
IFN-γ promoted the production of IL-10 and IL-6 induced by SEA from RAW 264.7 **cells.** (A) IL-10 production tested by ELISA in supernatants of RAW 264.7 cells stimulated with IFN-γ (4 ng/ml), 40 µg/ml of SEA (SEA40), or cultured with medium alone (untreated). (B) IL-10 production tested by ELISA in supernatants of RAW 264.7 cells stimulated with 5, 2.5, 1.25 µg/ml of SEA or cultured with medium alone. (C) IL-10 production tested by ELISA in supernatants of RAW 264.7 cells stimulated with 4 ng/ml of IFN-γ and serially diluted SEA from 5 µg/ml to 0.313 µg/ml simultaneously or RAW 264.7 cells cultured with medium alone. (D) IL-6 production in supernatants of RAW 264.7 cells stimulated with 40 µg/ml of SEA (SEA40), 4 ng/ml of IFN-γ, 0.65 µg/ml of SEA and 4 ng/ml of IFN-γ simultaneously (SEA0.65 + IFN-γ), 0.65 µg/ml of SEA (SEA0.65), and cultured with medium alone (untreated). (*P<0.05, **P<0.01, Duncan's multiple range test).

Without IFN-γ, 40 µg/ml and 5 µg/ml of SEA induced significant up-regulation of IL-10 production in RAW 264.7 cells comparing to non-SEA treatment (*P*<0.01, *P*<0.05 respectively) (Figure 2A and 2B). SEA concentration lower than 2.5 µg/ml failed to induce IL-10 from RAW 264.7 cells (Figure 2B). When IFN-γ is presented, SEA significantly attenuate IFN-γ-induced MHC class II expression even when its concentration was decreased to 0.625 µg/ml (Figure 1C).

SEA could significantly attenuate IFN-γ-induced MHC class II expression even when its concentration was decreased to 0.625 µg/ml (Figure 1C). To explore the underlying reasons responsible for attenuating IFN-γ-induced MHC class II expression by low concentration of SEA, we detected IL-10 production in supernatants of RAW 264.7 cells primed with SEA and IFN-γ simultaneously. Surprisingly, we found that although RAW 264.7 cells could only generate about 17 pg/ml of IL-10 with the stimulation of 5 µg/ml SEA alone for 48 h (Figure 2B), they could generate about 680 pg/ml of IL-10 when were stimulated simultaneously with 5 µg/ml SEA and IFN-γ for 48 h (Figure 2C). Moreover, SEA at 2.5, 1.25 and 0.65 µg/ml still possess the same effect as 5 µg/ml of SEA at inducing IL-10 production in the presence of IFN-γ (Figure 2C). When the concentration of SEA was decreased to 0.313 µg/ml, the IL-10 production induced by stimulation of SEA and IFN-γ simultaneously from RAW 264.7 cells was still kept in a relatively high level (about 450 pg/ml) although it showed a significant decrease when compared with that induced by 0.65 µg/ml of SEA and IFN-γ simultaneously (*P*<0.01) (Figure 2C). These results suggested that IFN-γ could enhance the ability of SEA at inducing IL-10 from RAW 264.7 cells.

Similarly, we tested the IL-6 production induced by high and low level of SEA in presence or absence of IFN-γ. 40 µg/ml of SEA induced significant up-regulation of IL-6 production from RAW 264.7 cells (*P*<0.01) (Figure 2D). 0.65 µg/ml of SEA alone failed to induce IL-6 from RAW 264.7 cells. However, when RAW 264.7 cells were stimulated simultaneously with 0.65 µg/ml of SEA and IFN-γ, they could generate significantly increased production of IL-6 (*P*<0.01) (Figure 2D). This suggested that IFN-γ could also enhance the ability of SEA at inducing IL-6 from RAW 264.7 cells.

### The Attenuation of SEA on IFN-γ-induced MHC Class II Expression was Mediated at least Partly by IL-10 and IL-6

**Figure 3:**
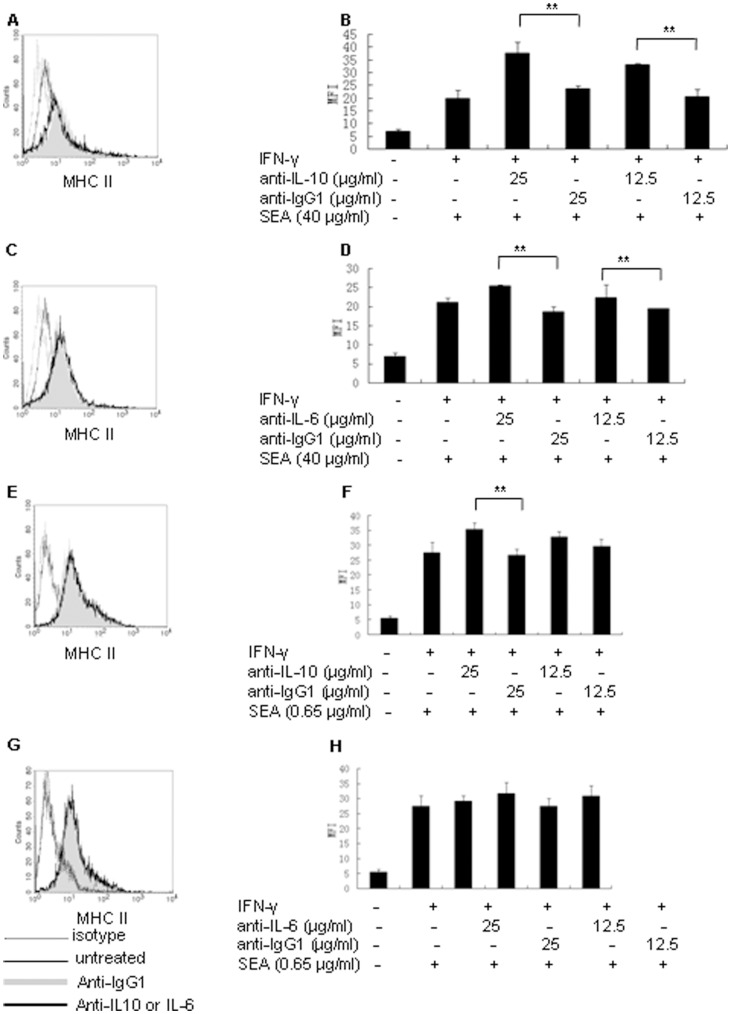
SEA attenuated IFN-γ-induced MHC class II expression in RAW 264.7 cells was mediated partly by IL-10 and IL-6. (A, C, E, G) Flow cytometry analysis of MHC class II expression in RAW 264.7 cells cultured with anti-IL-10 or anti-IL-6 antibody treated cell culture supernatants of RAW 264.7 cells stimulated simultaneously with IFN-γ and 40 µg/ml of SEA (A,C) or IFN-γ and 0.6 µg/ml of SEA (E, G). isotype (open histogram with dotted line), untreated (open histogram with thin line), antiIgG1 (gray histogram), anti-IL-10 (A, E) or anti-IL-6 (C, G) (open histogram with bold line). (B, D, F, H) Histogram of mean fluorescence intensity of MHC class II in RAW 264.7 cells cultured with anti-IL-10 or anti-IL-6 antibody treated cell culture supernatants of RAW 264.7 cells stimulated simultaneously with IFN-γ and 40 µg/ml of SEA (B, D) or with IFN-γ and 0.65 µg/ml of SEA (F, H). (**P<0.01).

To ascertain whether IL-10 and IL-6 induced by SEA from RAW 264.7 cells might mediate the inhibition of IFN-γ-induced MHC class II expression, we adopted antibody neutralization tests. In short, the supernatants of RAW 264.7 cells primed with 40 or 0.65 µg/ml of SEA and IFN-γ simultaneously were collected and then prepared for the antibody neutralization tests. The supernatants were then pretreated with anti-IL-10 or anti-IL-6 antibodies, or their isotype controls in the presence of IFN-γ for 2 h at 37°C. Fresh medium alone or fresh medium containing IFN-γ were used as control. Following, the pretreated supernatants and control medium were used for culturing RAW 264.7 cells for 48 h. At the end of culture, MHC class II expression was detected by flow cytometry. The results indicated that neutralization of IL-10 (Figure 3A, 3B) or IL-6 (Figure 3C, 3D) in the supernatants of RAW 264.7 cells primed with 40 µg/ml of SEA and IFN-γ simultaneously both resulted in significant up-regulation of MHC class II expression (*P*<0.01). Neutralization of IL-10 (Figure 3E, 3F) but not IL-6 (Figure 3G, 3H) in supernatants of RAW 264.7 cells primed with 0.65 µg/ml of SEA and IFN-γ simultaneously led to significant up-regulation of MHC class II expression. These results suggested that IL-10 might play a more important role than IL-6 in mediating the suppression of MHC class II expression by SEA.

### SEA Attenuated MHC Class II Expression through TLR4

**Figure 4:**
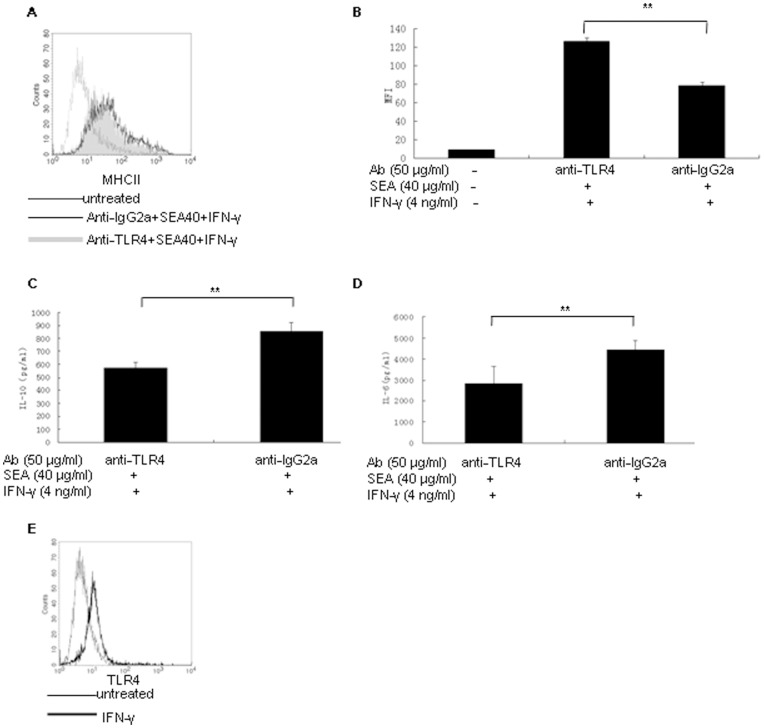
SEA attenuated MHC class II expression in RAW 264.7 cells through TLR4 and TLR4-induced IL-10 and IL-6. (A) Flow cytometry analysis of MHC class II expression in RAW 264.7 cells stimulated with 40 µg/ml of SEA and IFN-γ simultaneously in condition of being pretreated with anti-TLR4 antibody (gray histogram), anti-IgG2a antibody (open histogram with thin line) or cultured with medium alone (untreated, open histogram with dotted line). (B) Histogram of the MFI of MHC class II expression in RAW 264.7 cells stimulated with 40 µg/ml of SEA and IFN-γ simultaneously following blocked with anti-TLR4 or anti-IgG2a antibody. The cells cultured with medium alone were used as control. (C) IL-10 production tested by ELISA in supernatants of RAW 264.7 cells being blocked with anti-TLR4 antibody or anti-IgG2a antibody and then being stimulated simultaneously with SEA (40 µg/ml) and IFN-γ (4 ng/ml). (D) IL-6 production tested by ELISA in supernatants of RAW 264.7 cells being blocked with anti-TLR4 antibody or anti-IgG2a antibody and then being stimulated simultaneously with SEA (40 µg/ml) and IFN-γ (4 ng/ml). (**P<0.01). (E) Flow cytometry analysis of TLR4 expression in RAW 264.7 cells stimulated with IFN-γ (open histogram with bold line) or cultured with medium alone (open histogram with thin line).

Pattern recognition receptors play an important role in regulating immune response through initiation of cytokines production following interaction with pathogen-associated molecular patterns [2]. TLR4 is expressed on cell surface and is involved in recognition of the constituents contained in schistosome SEA [15]. To understand the role of TLR4 on attenuating MHC class II expression in RAW 264.7 cells by SEA, we adopted an antibody blocking assay. In short, after being incubated with anti-TLR4 monoclonal antibody or the isotype-matched control for 30 min at 37°C, these RAW 264.7 cells were incubated with 40 µg/ml of SEA and IFN-γ simultaneously for 48 h. Cells cultured in medium alone were used as control. The expression of MHC class II in these RAW 264.7 cells was detected by flow cytometry and the level of IL-10 and IL-6 in supernatants was assayed by ELISA. The results indicated that blocking TLR4 in RAW 264.7 cells leads to significant increase of MHC class II expression (*P*<0.01) (Figure 4A, 4B) and significant decrease in IL-10 (Figure 4C) and IL-6 (Figure 4D) production compared with the isotype control antibody treated one (*P*<0.01). These results suggested that TLR4 might play an important role in mediating SEA-induced production of IL-10 and IL-6, and the suppression of MHC class II expression in RAW 264.7 cells through interaction with some components contained in schistosome SEA. It has been suggested that the expression of pattern recognition receptors might be regulated by cytokines [16], [17]. In our experiments, we found that IFN-γ could up-regulate TLR4 expression in RAW 264.7 cells (Figure 4E). The increased expression of TLR4 in RAW 264.7 cells stimulated with IFN-γ might improve the ability of RAW 264.7 cells in recognizing the components contained in SEA and might represent a reasonable explanation for the significant increasing in IL-10 and IL-6 production in supernatants of RAW 264.7 cells stimulated with 0.65 µg/ml of SEA and IFN-γ simultaneously compared with that being stimulated with 0.65 µg/ml of SEA alone.

### SEA Attenuated IFN-γ-induced MHC Class II Expression in Mouse Peritoneal Macrophages

**Figure 5:**
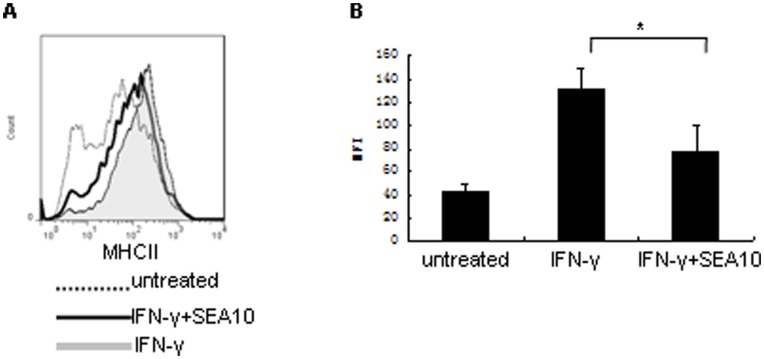
SEA attenuated MHC class II expression in primary macrophages. (A) Flow cytometric analysis of MHC class II expression in primary mouse peritoneal macrophages stimulated with 10 ng/ml IFN-γ (R&D) (shaded histogram), 10 µg/ml of SEA (SEA10, pretreated with endotoxin removing gel) and IFN-γ simultaneously (open histogram with thick line) and untreated cells (open histogram with dotted line). (B) Histogram showing the MFI of MHC class II expression in primary mouse peritoneal macrophages stimulated with IFN-γ, 10 µg/ml of SEA (SEA10, pretreated with endotoxin removing gel) and IFN-γ simultaneously (IFN-γ + SEA10) and cells cultured with medium alone (untreated). (* *P*<0.05).

To find out whether SEA attenuated IFN-γ-induced MHC class II expression can also happen in primary macrophages, we isolated mouse peritoneal macrophages and treated them with 10 ng/80 IU/ml IFN-γ (R&D) in presence or absence of 10 µg/ml SEA. The purity of mouse peritoneal macrophages reached 80% based on the results of flow cytometry tested with PE-conjugated rat anti-mouse F4/80 (data not shown). SEA significantly attenuated IFN-γ-induced MHC class II expression in mouse peritoneal macrophages (Figure 5A and 5B) (*P<0.05).

## Discussion

With the chronicity of schistosomiasis, host responsiveness to schistosome eggs is alleviated [18], [19]. It is manifested as the spontaneous diminution of the granuloma formation around the newly deposited schistosome eggs in the chronic stage of the disease, which was described as ‘endogenous desensitization’ in the 1960s [8]. Along with this, the host’s immune responses shifts from Th1 to Th2 phenotype accompanied with the up-regulation of a lot of cytokines including IL-10, TGF-β and IL-6 [14]. The immune response elicited by SEA around deposited eggs belongs to the delayed-type, T cell-mediated hypersensitivity [20], [21] which depends on MHC class II-mediated antigen presentation [22] and IFN-γ signaling [23].

Previous studies have suggested that the components contained in SEA might possess the ability to down-regulate immune responses. For example, egg-tolerized mice suffered high mortalities due to an excessive immune response that brought about lethal immune damage [24], SEA could successfully ameliorate and prevent the progression of experimental autoimmune encephalomyelitis (EAE) in mice [25]. By an *in vitro* analysis on RAW 264.7 cell line, our study contributes new information for understanding the negative regulation of SEA. We found that SEA could attenuate IFN-γ-induced MHC class II expression by inducing IL-10 and IL-6 production. Moreover, we found that SEA alone at a high level (40 µg/ml) could also induce high level of IL-10 and IL-6 from RAW 264.7 cells and that MHC class II expression in RAW 264.7 cells stimulated with 40 µg/ml SEA could be up-regulated significantly, which was much weaker than that induced by IFN-γ (data not shown). Based on the pivotal role of the expression of MHC class II in initiation and regulation of immune responses, we could deduce that schistosome might suppress the host’s immune response through down-regulating MHC class II expression to evade immune attack. This finding supports a viewpoint derived from a study conducted in *Schistosoma mansoni* infection that showed I-A expression on granuloma macrophages was regulated by SEA or SEA stimulated factors [6].

Antigen presenting cells recognize pathogen-associated molecular patterns through pattern recognition receptors, which resulted in inflammatory cytokines gene expression [2]. Phagocytosis-mediated antigen presentation together with TLR-mediated inflammatory cytokines expression instructs the development of antigen-specific adaptive immunity [2]. Our study showed that SEA down-regulated the expression of MHC class II in RAW 264.7 cells through IL-10 and IL-6 induction. These results supported that pathogens could down-regulate immune responses by inducing IL-10 [12] or IL-6 [13] which could attenuate MHC class II expression through negative feedback effects. The ability of SEA to induce IL-10 was stronger than that of IL-6. Our results showed that the production of IL-10 from RAW 264.7 cells stimulated with 0.65 µg/ml of SEA and IFN-γ was nearly equal to that with 40 µg/ml of SEA. IL-10 play the major role in mediating the down-regulation of MHC class II expression in RAW 264.7 cells as our results showed that neutralization of IL-10 but not IL-6 in supernatants of RAW 264.7 cells stimulated with 0.65 µg/ml of SEA and IFN-γ simultaneously could lead to the up-regulation of MHC class II. IL-10 and TGF-β were both induced and both recognized as factors can promote host survival by suppressing pro-inflammatory cytokine production and ova-induced hepatotoxicity in the acute phase of schistosomiasis [26]. Anti-inflammatory effects of IL-10 are well known including down-regulation of IFN-γ-induced MHC class II expression [12], [27]. Although TGF-β was reported playing a role in suppressing both constitutive and cytokine-inducible MHC class II expression in macrophages [28], it could not play an important role in mediating the suppression of MHC class II expression in RAW 264.7 cells by SEA because IFN-γ show a suppressive effect on TGF-β induction as we observed in our study (data not shown). 40 µg/ml of SEA could induce 1800 pg/ml production of TGF-β from RAW 264.7 cells following the stimulation for 48 h (data not shown). However, TGF-β showed no significant change in supernatants of RAW 264.7 cells stimulated simultaneously with 0.65 µg/ml of SEA and IFN-γ compared with that produced by cells cultured with medium alone or stimulated with 0.65 µg/ml of SEA alone (data not shown). TGF-β production induced with 40 µg/ml of SEA and IFN-γ simultaneously from RAW264.7 cells decreased significantly compared with that were induced with 40 µg/ml of SEA alone (*P*<0.05) (data not shown). So the immunosuppressive effect of TGF-β in controlling the pathogenesis of schistosomiasis could be exerted maily through induction of Foxp3+ Treg cell [29] which was shown to be responsible for the down-regulation of granuloma inflammation [30], [31].

In innate immunity, suppressor of cytokine signaling (SOCS) proteins inhibit JAK/STAT signaling by various mechanisms [32]. The biological effects of IFN-γ are mediated mainly through a pathway in which STAT1 is the predominant transcription factor [33]. IL-10 and IL-6 engage receptors and then activate STAT3 and both induce the activation of SOCS3 which may differently modulate STAT3 activation induced by IL-10 and IL-6 [34]. In this paper, we showed that SEA can suppress the expression of MHC class II at transcriptional level and this effect is related with the IL-10 and IL-6 released from SEA activated macrophages. Whether *Schistosoma japonicum* SEA can result in transcriptional up-regulation of SOCS3 and thus inhibit IFN-γ-induced phosphorylation of STAT-1 and transcription of CIITA in SEA-stimulated macrophages via a mechanism involving SOCS3 need to be studied further.

SOCS3 can be recruited by IL-6 signal transducer gp130 to its phosphotyrosine residues (Y759), but not by the phosphorylated tyrosine motifs of the IL-10R [34]. The difference between the IL-10 and IL-6 induced by SEA from macrophages might be related with the different sensitivities of IL-10 and IL-6 signaling toward mechanisms that inhibit the Janus kinase/STAT pathway.

A major concern about the effect of SEA on MHC class II expression is the involvement of the possible contaminated endotoxin in SEA. In order to exclude the possible effect of endotoxin, we treated SEA repeatedly with endotoxin removing gel and then tested the endotoxin level in the prepared SEA by Tachypleus Amebocyte Lysate (TAL) kit. The results showed that following the treatments there was no contaminated endotoxin contained in the SEA used for our experiments. These observations could exclude the possibility that the effects of SEA on RAW 264.7 cells observed in our study was due to contaminated endotoxin.

The expression of MHC class II might be regulated at multiple segments [35]. As a master controller of MHC class II gene activation, the transactivator CIITA play a pivotal role in control of cellular immune responses through quantitative regulation of MHC class II expression [11]. Our study showed that SEA could significantly attenuate CIITA transcription induced by IFN-γ, suggesting that the attenuation of MHC class II expression by SEA might occur at transcriptional level. This result is supported by gene microarray information analysis based on an *in vivo* experiment by use of cells derived from *Schistosoma japonicum* infected mouse livers obtained from various stages of the infection. The transcription level of histocompatibility 2, class II antigen including H2-Aa, H2-Ab1, H2-Ob, H2-Eb and H2-Ea changed consistently with the development of mouse schistosomiasis from 0–18 weeks, that is, up-regulated at 6 weeks (acute stage) and then down-regulated until the 18^th^ week post-infection (chronic stage) (data not shown). These results strongly suggested that MHC class II expression was regulated in association with egg deposition and SEA release. Our experiments with mouse peritoneal macrophages showed that the attenuation on macrophages MHC class II expression level by SEA not only occured in RAW 264.7 cells but also occured in primary macrophages. These results strongly suggested that MHC class II expression was regulated in association with egg deposition and SEA release.

Expression of class II molecules is exquisitely controlled at the transcriptional level. CIITA, which does not bind directly to the promoter, is a master controller among the large set of proteins interact with the promoters of class II genes [36]. In our research, we found that 0.65 µg/ml of SEA completely suppressed IFN-γ induced CIITA expression (Figure 1D), however, in presence of 0.65 µg/ml of SEA, the IFN-γ induced MHC class II still showed an significant up-regulation compared with the untreated cells (Figure 1C). This suggested that after being stimulated with IFN-γ and 0.65 µg/ml of SEA simultaneously for 48 h, although CIITA has been suppressed completely on the transcriptional level, the protein of MHC class II expressed on the cell surface was still not experienced degradation totally. It was reported that in immature dendritic cells (DCs), peptide loaded MHC II (MHC II-p) is ubiquitinated after peptide loading and thus driving its sorting to the luminal vesicles of multivesicular bodies. These luminal vesicles and the MHC II-p they carry, are delivered to lysosomes for degradation. In DCs that are activated by pathogens or inflammatory stimuli, peptide loaded MHC II is inefficiently ubiquitinated and thus allowing its transfer to and stable expression at the plasma membrane [37]. Our results suggested that MHC class II might be inefficiently ubiquitinated and thus allowing its transfer to and stable expression at the plasma membrane of RAW 264.7 cells activated with SEA and IFN-γ for 48 h just as what happened in DCs as reported [37].

It has been shown that the expression of pattern recognition receptors might be regulated by cytokines [16], [17]. In our study, we found that IFN-γ could up-regulate TLR4 expression level in RAW 264.7 cells. Helminth carbohydrate lacto-*N*-fucopentaose III expressed by *Schistosoma mansoni* eggs could drive DC2 maturation through signaling via TLR4 [15]. Taken together, our results suggested that SEA might down-regulate MHC class II expression in RAW 264.7 cells through TLR4 signaling and TLR4-mediated IL-10 and IL-6 production. These results supplied additional evidence that TLR4 might mediate immunosuppression although more studies suggested that TLR4 mediated pro-inflammatory immune responses [38], [39].

## Materials and Methods

### Reagents

Dulbecco’s modified Eagle’s medium (DMEM) (Gibco, Grand Island, NY) was supplemented with 4.5 g/L D-Glucose, 2 mM L-glutamine, 10% heat-inactivated fetal bovine serum (PAA laboratories, Linz, Austria), 100 U of penicillin per ml, and 100 µg of streptomycin per ml (complete DMEM medium) before use. Recombinant mouse IFN-γ were purchased from PeproTech (London, UK; Cat. number is 315-05, 1 µg = 5×10^3^ IU) and R&D System (Cat. number is 485-MI, 1 µg = 8.43×10^3^ IU, which was only used in experiments including primary mouse peritoneal macrophages and RAW 264.7 cells stimulated with SEA pretreated with endotoxin removing gel). Phycoerythrin (PE)-conjugated rat anti-mouse MHC class II (I-A/I-E) (M5/114.15.2), PE-conjugated rat IgG2b isotype control, functional grade purified antibodies including anti-mouse interleukin-10 (IL-10) (JES5-2A5), anti-mouse interleukin-6 (IL-6) (MP5-20F3) and their isotype control rat IgG1, anti-mouse Toll-like receptor 4 (TLR4)/MD-2 (MTS510) and its isotype control rat IgG2a were all from eBioscience (San Diego, CA, USA). PE anti-mouse F4/80 antibody, FITC anti-mouse I-A/I-E antibody and FITC rat IgG2b isotype control were from Biolegend. ELISA kit including mouse IL-10 and mouse IL-6 were all from Bender MedSystems (Vienna, Austria). TRizol Reagent for preparing intact RNA were from Invitrogen (Carlsbad, CA, USA). Super RT Kit for cDNA first strand synthesis was from BioTeke (Beijing, China). SYBR Premix Ex Taq™ (Perfect Real Time) were from TaKaRa (Kyoto, Japan). Detoxi-Gel™ Endotoxin Removing Gel were purchased from Thermo Scientific Pierce. The endotoxin testing kit were from Zhanjiang Bokang Marine Biological Co., LTD (Guangdong, P.R.China).

**Table 1:** Primers used for real-time PCR.

*Gene*	*Primer Sequence*	*Size (bp)*
CIITA	5′-acacctggacctggactcac-3′	229
	5′-gctcttggctcctttgtcac-3′	
β-actin	5′-ggaaatcgtgcgtgacatc-3′	180
	5′-aaggaaggctggaaaagagc-3′	

### Preparation of SEA

*Schistosoma japonicum* eggs were isolated from infected liver and intestine of rabbit. Purified eggs in PBS with protease inhibitor cocktail were sonicated three times on ice for 10 min. The suspension was freeze-thawed several times and centrifuged at 30,000 g for 30 min at 4°C. The supernatant was passed through a 0.22 µm filter and used as soluble egg antigen (SEA). Protein concentration was determined by BCA protein Assay Kit (Pierce Biotechnology, Inc., IL, USA) [40]. To exclude the effects of endotoxin, SEA was repeatedly treated with Detoxi-Gel™ Endotoxin Removing Gel (Thermo Scientific Pierce) before being used.

### Testing Endotoxic Activity by Gel-clot TAL Test

Materials required for TAL test including TAL, control standard endotoxin (10 EU/ml), TAL reagent water, test tubes and pipette tips free of detectable endotoxin. The gel-clot TAL tests were preformed according to the method described in Chinese Pharmacopoeia 2010 edition. Briefly, 100 µl series dilutions of control standard endotoxin were mixed with 100 µl TAL reagents. The mixed solutions were incubated at 37°C for 60 minutes. The formation of the gel was scored by turning each test tube upside down. If the gel remained a piece, it is considered a solid gel formation. Otherwise, it is considered a failed gel formation.

### Cell Culture

Murine RAW 264.7 macrophage cell line obtained from ATCC (ATCC TIB-71™, Manassas, VA) was maintained in complete DMEM medium. Cells were adjusted to a concentration of 1×10^6^/ml in complete medium for use in all of the cellular assays. To ascertain the appropriate concentration of IFN-γ used for up-regulation of MHC class II expression, RAW 264.7 cells were incubated with serially diluted IFN-γ for 48 h (4 ng/ml was selected as work concentration for IFN-γ from PeproTech Inc.). To observe the effect of SEA on MHC class II expression, RAW 264.7 cells were grown for 48 h in culture medium alone, SEA (40 µg/ml) or SEA (40 µg/ml) plus IFN-γ. To observe the dose-response relationship between SEA and MHC class II expression, RAW 264.7 cells were primed with SEA diluted serially from 40 µg/ml up to 0.156 µg/ml in presence of IFN-γ for 48 h. Cells cultured with medium alone or with IFN-γ were used as control. All these experiments were performed in 96-well plates, 200 µl per well. Cells were collected for detection of MHC class II expression after 48 h. In the experiments performed on RAW 264.7 cells with SEA pretreated with endotoxin removing gel or in the experiments performed on primary peritoneal macrophages, RAW 264.7 cells or primary macrophages were cultured with IFN-γ purchased from R?D System (10 ng/ml was selected as working concentration) with or without SEA (10 µg/ml was selected as working concentration, pretreated with endotoxin removing gel). For primary macrophages experiments, cells were plated in 24-well plate with complete medium, 6×10^5 ^per well. 12 hrs later, cells were respectively treated with 10 ng/ml IFN-γ (R&D System) in presence or absence of 10 µg/ml of SEA (pretreated with endotoxin removing gel) for 48 hrs. Cells cultured with medium alone were used as control.

### Preparation of Mouse Peritoneal Macrophages

C57BL/6 mice aged from 6 to 8 weeks were obtained from Shanghai Laboratory Animal Co. Ltd. (SLAC) (Shanghai, China). Peritoneal macrophages were recovered by peritoneal lavage with 5 ml of cold PBS and isolated by centrifugation. For adherent cells, cells (1×10^6^) were plated in 10 cm tissue culture dishes and cultured in RPMI 1640 medium containing 10% FBS, 100 U/ml penicillin and 100 µg/ml streptomycin for 2 h at 37°C, 5% CO_2_. Then medium was removed, and the non-attached cells were washed away with PBS and the remaining attached cells were used as peritoneal macrophages. PE rat anti-mouse F4/80 antibody were used for confirming the purity of the peritoneal macrophages.

### Cell Surface Staining and Flow Cytometry

RAW 264.7 cells were treated as described above. After 48 h, cells were recovered by being treated with 0.25% trypsine-EDTA for 10 min, terminated with DMEM containing 10% heat-inactivated FBS, washed with cold PBS for three times, and centrifuged at 400 *g*. RAW 264.7 cells were incubated with 5% bovine serum albumin in PBS on ice for 10 min and then incubated with phycoerythrin-conjugated rat anti-mouse MHC class II or TLR4 or phycoerythrin-conjugated rat IgG2b or IgG2a isotype control for 30 min on ice. In the experiments performed on RAW 264.7 cells with SEA pretreated with endotoxin removing gel, or experiments performed on primary peritoneal macrophages, cells were collected following stimulation for 48 h and labeled with FITC rat anti-mouse I-A/I-E (RAW264.7 cells) or double labeled with FITC rat anti-mouse I-A/I-E and PE rat anti-mouse F4/80 antibodies (primary macrophages). Then the cells were fixed with 1% paraformaldehyde and analyzed with a FACScan flow cytometer (Becton Dickinson), using CellQuest software (Becton Dickinson). Ten thousand events were recorded.

### Antibody Blocking Assay

The receptor responsible for recognition of SEA and mediate the production of IL-10 and IL-6 were detected using an antibody-blocking assay with anti-TLR4 antibody. RAW 264.7 cells were incubated in 96-well flat-bottom plates (2.0×10^5^ cells/well). For experiments blocking TLR4, adherent RAW 264.7 cells were treated with 50 µg/ml of anti-mouse TLR4 antibody or isotype control antibody IgG2a for 30 min at 37°C. Following incubation with antibodies, IFN-γ and SEA (40 µg/ml) were added and cells were incubated for 48 h at 37°C, 5% CO_2_. Then these cells were collected and assayed for MHC class II expression on cell surface. The culture supernatants were collected and assayed for IL-10 and IL-6 production. Cells cultured with medium alone were used as controls.

### Evaluation of Cytokines Production

IL-10 and IL-6 levels in culture supernatants were determined by ELISA using paired cytokine-specific monoclonal antibodies according to the manufacturer’s instructions.

### RNA Extraction and Reverse Transcription

RAW 264.7 cells were primed for 48 h with medium, SEA (0.65 µg/ml) in presence or absence of IFN-γ or IFN-γ alone in 60 mm dishes at 5.0×10^6^ cells/dish. Then total RNA was extracted according to the TRizol RNA isolation protocol. The extracted total RNA was dissolved in diethyl pyrocarbonate-treated water and subjected to reverse transcription using a Super RT kit, according to the manufacturer’s instructions, to synthesize the cDNA templates for real-time PCR analysis. Briefly, 100 ng of total RNA and Moloney murine leukemia virus reverse transcriptase were used to reverse transcribe RNA into cDNA at 42°C for 40 min.

### Real-time PCR Analyses

Real-time PCR was conducted using a commercially available SYBR Green I real-time PCR premix reagent containing Taq DNA polymerase and SYBR-Green I. cDNA templates and primers were added to the SYBR Green I PCR mix containing SYBR Premix Ex Taq, dNTP mixture, Mg^2+^ and SYBR Green I to give a total reaction volume of 25 µl. Real-time PCR reactions were then performed using an Applied Biosystems 7300 Real-time PCR System. Conditions were set to the following parameters: 30 s at 95°C, followed by 40 cycles at 95°C for 5 s, 60°C for 31 s; then 95°C for 15 s, 60°C for 1 min and 95°C for 15 s for the final dissociation stage to generate a melting curve for verification of amplification product specificity. The β-actin was used as an internal control under the same conditions. To obtain relatively accurate results, each template was processed in three tubes in the same PCR mixture. After the completion of PCR amplification, a melting curve analysis was performed. The 2^−ΔΔCt^ method [41] was used to analyze the relative changes in target gene transcription. The primer pairs used in real-time PCR analysis are listed in Table 1.

### Effect of IL-10 and IL-6 on MHC Class II Expression

To observe the effect of endogenous IL-10 and IL-6 on MHC class II expression, antibody neutralization test to supernatants of macrophages primed with SEA (0.65 µg/ml or 40 µg/ml) and IFN-γ simultaneously were performed in 96-well cell culture plates according to the reagent instructions. In brief, cell culture supernatants were collected after 48 h. Functional grade anti-IL-10 and anti-IL-6 antibodies or their respective isotype controls were serially diluted in 50 µl complete DMEM medium by 2-fold in the plates. Supernatants and IFN-γ were added to the culture plates, 100 µl per well. Simple medium or 50 µl medium plus 100 µl supernatants in presence of IFN-γ were used as controls. Following, the culture plates were incubated at 37°C, 5% CO_2_ for 2 h. RAW 264.7 cells were collected and adjusted to a density of 4×10^6^/ml. 50 µl of cell suspension was added to each well and cultured at 37°C, 5% CO_2_ for 48 h. Then the cells were harvested to determine MHC class II expression by flow cytometry. The results were indicated as mean fluorescence intensity to assay the MHC class II expression on cell surface.

### Statistical Methods

Data analysis was performed using software package SPSS for windows version 11.5 (SPSS Inc., Chicago, U.S.A). Results were presented in histograms with arithmetic mean ± SD (standard deviation). Comparisons of arithmetic means between two groups were analyzed using Student’s *t*-test. Analysis of variance (ANOVA test) was used to test the difference of arithmetic means among three or more groups. Least Significant Difference (LSD) method was used for multiple comparisons between groups. Linear regression was performed for evaluating dose-dependent effect. Differences between groups were considered to be statistically significant at the level of *P*<0.05. In all figures, * was used to indicate *P*<0.05 and ** for *P*<0.01. Data shown were from one representative experiment of three independent experiments. The results are comparable among the three experiments.
